# Categorical and dimensional aspects of stimulant medication effects in adult patients with ADHD and healthy controls

**DOI:** 10.3389/fphar.2024.1412178

**Published:** 2024-07-10

**Authors:** Per Thunberg, Maria Reingardt, Julia Rode, Mussie Msghina

**Affiliations:** ^1^ Center for Experimental and Biomedical Imaging in Örebro (CEBIO), Faculty of Medicine and Health, Örebro University, Örebro, Sweden; ^2^ Department of Radiology and Medical Physics, Faculty of Medicine and Health, Örebro University, Örebro, Sweden; ^3^ Department of Psychiatry, School of Medical Sciences, Faculty of Medicine and Health, Örebro University, Örebro, Sweden; ^4^ Centre for Clinical Research and Education, Central Hospital, Karlstad, Sweden; ^5^ School of Medical Sciences, Faculty of Medicine and Health, Örebro University, Örebro, Sweden; ^6^ Department of Clinical Neuroscience, Karolinska Institutet, Stockholm, Sweden

**Keywords:** cognitive control, central stimulants, ADHD, AX-CPT, proactive behavioral index

## Abstract

Psychiatric disorders are categorized on the basis of presence and absence of diagnostic criteria using classification systems such as the international classification of diseases (ICD) and the diagnostic and statistical manual for mental disorders (DSM). The research domain criteria (RDoC) initiative provides an alternative dimensional framework for conceptualizing mental disorders. In the present paper, we studied neural and behavioral effects of central stimulant (CS) medication in adults with attention deficit hyperactivity disorder (ADHD) and healthy controls using categorical and dimensional stratifications. AX-Continuous Performance Task (AX-CPT) was utilized for the later purpose, and participants were classified as “reactive” or “proactive” based on their baseline proactive behavioral index (PBI). Out of the 65 individuals who participated (33 healthy controls and 32 patients with ADHD), 53 were included in the final analysis that consisted of 31 healthy controls and 22 ADHD patients. For the dimensional stratification, a median split of PBI scores divided participants into “reactive” and “proactive” groups irrespective of whether they had ADHD or not. Participants performed AX-CPT in conjunction with functional magnetic resonance imaging (fMRI) before and after CS medication. We found no significant within or between group CS effect when participants were categorically assigned as healthy controls and ADHD patients. For the dimensional stratification, however, CS selectively increased activation in frontoparietal cognitive areas and induced a shift towards proactive control mode in the reactive group, without significantly affecting the proactive group. In conclusion, the neural and behavioral effects of CS were more clear-cut when participants were stratified into dimensional groups rather than diagnostic categories.

## 1 Introduction

Psychiatric disorders, as a rule complex syndromes with substantial heterogeneity, are categorized into distinct diagnostic groups on the basis of presence and absence of characteristic criteria using classification systems such as the diagnostic and statistical manual for mental disorders (DSM) (American Psychiatric Association, 2013) and the international classification of disease (ICD). The research domain criteria initiative (RDoC) takes a different approach and utilizes neurobiologically informed domains of function to provide a dimensional framework for conceptualizing mental disorders (Cuthbert, 2022). In clinical settings, DSM/ICD classifications are used exclusively. However, these classification systems lead to widely heterogeneous groups within the same diagnostic category and their utility for research purposes has been questioned (Widiger and Samuel, 2005; Wardenaar and de Jonge, 2013; Borgogna, Owen, and Aita, 2023). In the present study, we compared effects of stimulant medication in healthy individuals and adults with attention deficit hyperactivity disorder (ADHD) using categorical (presence and absence of ADHD diagnosis according to DSM 5) and dimensional (RDoC cognitive control construct) stratification methods.

ADHD is a neurodevelopmental disorder, with impaired cognitive control and impulsivity/hyperactivity as core symptoms (Faraone et al., 2021). Although not part of the diagnostic criteria, there is also evidence indicating that emotional dysregulation may be a core impairment in ADHD, making ADHD an even more heterogeneous syndrome than currently clinically conceptualized (Retz et al., 2012; Shaw et al., 2014). Central stimulants (CS), which act by blocking dopamine (DA) and noradrenaline (NA) reuptake, are the mainstay of pharmacological treatment (Volkow et al., 2001; Arnsten and Dudley, 2005; Berridge et al., 2006; Faraone and Glatt, 2010), and their effect in alleviating core ADHD symptoms has been shown to be robust in the clinical setting (Faraone and Glatt, 2010; Cortese et al., 2018). Roughly 70%–80% of ADHD patients treated with CS are clinically deemed to be treatment responders (Spencer et al., 2005). CS is also used by segments of the healthy population wanting to improve cognitive performance, but the beneficial effects of CS in normal functioning adults is less clear (Repantis et al., 2010; Smith and Farah, 2011). Meta-analyses in healthy individuals showed small but significant effects of methylphenidate (MPH) on working memory, inhibitory control and processing speed (Smith and Farah, 2011; Marraccini et al., 2016). A meta-analysis that compared the effect of three cognitive enhancers in healthy individuals, MPH, modafinil and dexamphetamine, found that MPH had the strongest effect of the three, with small improvements on recall, attention and inhibitory control (Roberts et al., 2020). A recent study that evaluated the effects of CS in everyday complex tasks in healthy subjects showed that CS increased effort but reduced quality of effort, and performance across participants was reversed by CS such that above average performers ended up being below average after CS and vice versa for below average performers (Bowman et al., 2023). Effects of CS have also been shown to vary with baseline cognitive capacity (Mattay et al., 2000; Cools et al., 2008; Rostami Kandroodi et al., 2021) and baseline DA and NA levels (Cools and D'Esposito, 2011). Thus, not only effects of CS may differ between healthy subjects and ADHD patients, but they may also differ within the same group on the bases of baseline behavioral and neurochemical factors.

Cognitive control, also known as top-down control or executive control is a neuropsychological construct pertaining to the flexible regulation of goal-directed behaviour and is generally associated with the functions of lateral prefrontal cortex and posterior parietal cortex (Miller and Cohen, 2001; Friedman and Robbins, 2022). Cognitive control subconstructs such as goal selection, updating, representation, and maintenance can be studied using AX-Continuous Performance Task (AX-CPT), a paradigm that is often used to assess cognitive control (Lopez-Garcia et al., 2016). In AX-CPT, single letters (A, B, X, Y) are displayed on a screen and participants are instructed to make a target response when presented with the letter X, but only if it is preceded by the letter A. For all other letter combinations, participants are instructed to make a non-target response. To create expectancy, AX trials are generally made to occur more frequently than AY, BX and BY trials (Barch et al., 2003). Cognitive control mode during AX-CPT is assessed by the proactive behavioral index (PBI), where a proactive cognitive control mode indicates better performance on BX trials than AY trials, and the opposite being the case for reactive control mode.

In a study that made use of the dual system theory of decision-making that contrasts quick heuristic mode of decision making with a slower deliberative mode, Yechiam and Zeif found that MPH improved performance by selectively enhancing the slower deliberative mode of decision-making compared to the quicker heuristic mode (Yechiam and Zeif, 2022). A conceptually comparable dual mechanism framework (DMC) has been suggested for cognitive control, contrasting proactive and reactive control modes (Braver et al., 2009; Braver, 2012). The proactive control mode has been shown to dominate in healthy adults, while patients with psychotic (Barch et al., 2003; MacDonald and Carter, 2003) and anxiety disorders (Schmid, Kleiman, and Amodio, 2015) are more prone to employ a more reactive control mode.

In the present paper, we studied behavioral and neural effects of CS in healthy controls and individuals with ADHD using two stratification strategies, (i) categorical stratification on the bases of presence and absence of ADHD diagnosis according to DSM 5, and (ii) dimensional stratification on the basis of proactive and reactive cognitive control mode. We hypothesized that the behavioral and neural effects of CS in the categorical and dimensional groups may not always parallel each other, and that the neurobehavioral homogeneity created by dimensional stratification using RDoC cognitive control domain might reveal CS effects masked by heterogeneity in the categorical stratification using diagnosis classes.

## 2 Materials and methods

The study was approved by the Swedish Ethical Review Authority (Dnr 2020-02278; 2020-05590) and written consent was obtained from all participants in the study.

### 2.1 Participants and study outline

Thirty-three healthy controls and 32 adults with ADHD were included in the study that was conducted between December 2020 and December 2023. The ADHD group was a well-characterized clinical cohort, who had clinically responded to CS medication and are recruited from the Neuropsychiatric Outpatient Clinic at Örebro University Hospital (Rode et al., 2023). Inclusion criteria for the ADHD cohort were (i) ADHD diagnosis after extensive neuropsychiatric evaluation by a dedicated team of psychologists and senior consultants in psychiatry according to Swedish guidelines, (ii) no ongoing psychosis, bipolar, depressive, substance use or sever autism spectrum disorder, (iii) no suicidal or aggressive tendency and (iv) no contraindication for MRI investigation. Choice of medication and treatment optimization was carried out by the treating physician and followed Swedish guidelines for the pharmacological treatment of ADHD (Läkemedelsverket, 2016). Patients had to have a minimum of 4–6 weeks of stable medication before they could be enrolled in the study. Clinical response was determined by a score of 1 or 2 using clinician and patient-rated Clinical Global Impression–Improvement (CGI-I). The control group was recruited by advertising at a university campus and hospital area. Exclusion criteria for the healthy controls were (i) current or previous psychiatric and neurological ailment including substance use, (ii) ongoing psychoactive medication use, (iii) narrow-angle glaucoma, and (iv) incompatibility with magnetic resonance imaging (MRI). The control group was also assessed for potential allergic reaction to MPH or any of its ingredients. The CS medication used by the ADHD patients was either methylphenidate (MPH) or lisdexamfetamine (LDX) as selected and dose-optimized by the treating physician.

All participants underwent MRI examinations before and after CS using the same MRI scanner and protocol settings. The first session was performed in the absence of CS and the second 1–2 h after ingestion of CS, which constituted of 30 mg short-acting MPH for the healthy controls and MPH or LDX for the ADHD group as selected and dose-optimized by the treating physician. ADHD patients were instructed to abstain from CS medication for 24 h before the start of the first MRI session. CS was ingested directly after the end of the first session, and the second session started 1–2 h later to synchronize MRI examination with peak CS concentration in brain tissue. Each MRI session included a functional MRI (fMRI) acquisition with the participants performing an AX-CPT task that lasted for 14 min and 22 s.

### 2.2 Cognitive control task (AX-CPT)

AX-CPT was implemented using E-Prime (Psychology Software Tools version 3.0, Pittsburgh, PA, United States) and started with a rest period of 30 s displaying a fixation cross at the center of the screen, followed by 160 AX-CPT trials. An AX-CPT trial consisted of two stimuli; a cue letter (“A” or “B”) followed by a probe letter (“X” or “Y”) with a blank inter-stimulus interval (ISI) in between, followed by an inter-trial interval (ITI) where a fixation cross was displayed. Participants used pistol-grips held in each hand and target-responses were made by pressing a button on the right grip and on the left grip for non-target responses. Reaction time (RT) and response were recorded from the onset of the probe to, at maximum, the end of the ITI. Duration of cue and probe was 500 msec. ISI was jittered between 900–1,100 msec with average duration of 1,000 msec and ITI was jittered between 1,500–2,500 msec with average duration of 2,000 msec. The order of the trial types was randomized, with 70% being target trials (AX) and 30% divided equally between non-target trials (AY, BX, BY). Participants got instructions about AX-CPT and practiced prior MRI until they felt sure they understood the task and could perform it with ease.

RT and responses were analyzed separately for each trial type (AX, AY, BX and AY). The proactive behavioral index (PBI) was calculated using the RTs for AY and BX according to the formula (AY-BX)/(AY + BX) (Braver et al., 2009; Gonthier et al., 2016). High PBI was interpreted as a dominance of a proactive cognitive control mode and low PBI as dominance of reactive control mode. The calculation of error rate for the different trial types was based on recorded responses (correct/incorrect) while trials without any responses were omitted.

### 2.3 Categorical and dimensional stratification

Participants were stratified into categorical and dimensional groups on the basis of presence and absence of ADHD diagnosis according to DSM 5 (categorical stratification), or on the basis of RDoC cognitive control domain (dimensional stratification). For the categorical stratification, the two groups thus consisted of healthy controls and ADHD patients, and for the dimensional stratification of reactive and proactive individuals irrespective of their DSM 5 diagnosis status. The two dimensional groups (reactive vs. proactive) were created by a median split of baseline PBI scores (PBI_pre_). Participants with PBI_pre_ score less than the median value formed the reactive group and those with PBI_pre_ score equal to or greater than the median value formed the proactive group.

### 2.4 MRI and preprocessing of fMRI data

A 3.0T MR system (Signa Premier, GE Medical Systems, WI) and a 48-channel head coil were used for all MR examinations, which included a structural image of the brain and an fMRI acquisition during the AX-CPT task. Parameters for the structural scan (3D T1w IR-prepared fast spoiled gradient recalled echo, “BRAVO”) were: TR/TE = 7.3/3.0 msec, acquired voxel size 0.9 × 0.9 × 1.2 mm, parallel imaging acceleration (ARC) factor of 2. The fMRI acquisition used a gradient echo EPI pulse sequence with TR/TE = 2000/35 msec, slice thickness 2.5 mm, acquired pixel size 2.5 × 2.5 mm, no slice gap, ARC factor of 2 and a hyperband factor of 2. Acquired images were converted to nifti-format using dcm2niix (https://github.com/rordenlab/dcm2niix).

Preprocessing of fMRI data (slice-time correction, realigning, unwarping, normalizing to MNI template, smoothing) was performed using CONN (Whitfield-Gabrieli and Nieto-Castanon, 2012). Default settings were applied except for a smoothing kernel of 6 mm instead of 8 mm. The final preprocessing step included anatomical component-based noise correction (aCompCor) based on white matter and cerebrospinal fluid (CSF), scrubbing and bandpass filtering. Further details regarding the preprocessing are provided in the supplement.

### 2.5 fMRI analysis

First-level analysis of each AX-CPT acquisition (pre and post CS) was performed using a general linear model (GLM) consisting of 4 regressors (AX, AY, BX and BY trials) where the onset and duration for each trial started with the presentation of the cue and lasted until the end of ITI. Group (second-level) analysis was carried out by setting up a full factorial model including beta values (i.e., BOLD signals) from the four different trials (AX, AY, BX and BY), before and after the administration of CS.

Correlations were tested between baseline PBI (PBI_pre_) and differences in brain activity pre and post CS for the trial types (AY, BX). This was achieved by calculating a difference image (BOLD image post—BOLD image pre) for each trial type and then correlating it using PBI_pre_ as covariate.

Exploratory whole-brain analysis was performed and to correct for multiple comparisons, a pixelwise significance threshold level of p < 0.001 and cluster size threshold of FWE<0.05 were applied. All fMRI analyses were performed using SPM12 running on Matlab R2019b.

Labels in the Harvard-Oxford cortical structural atlas, available in FSLeyes (version 0.34.2, https://git.fmrib.ox.ac.uk/fsl/fsleyes/fsleyes), were used to provide anatomical information about significant clusters.

### 2.6 Statistical analysis

Behavioral data were analyzed using SPSS (IBM SPSS Statistics version 28.0), and Matlab (R2019b) was used for graphical plots. Participants with PBI_pre_ outside the range [Q1-1.5xIQR, Q3+1.5xIQR] were considered as outliers and their experimental protocol was reviewed for any outstanding issues that may explain the extreme values. (Q1 = 25th percentile, Q3 = 75th percentile, interquartile range (IQR) = Q3-Q1). Parametric tests were applied if the data could be adequately modelled according to the normal distribution, otherwise non-parametric tests were used. Two-sided p-values are reported in all cases.

## 3 Results

### 3.1 Study groups and participant data

**FIGURE 1:**
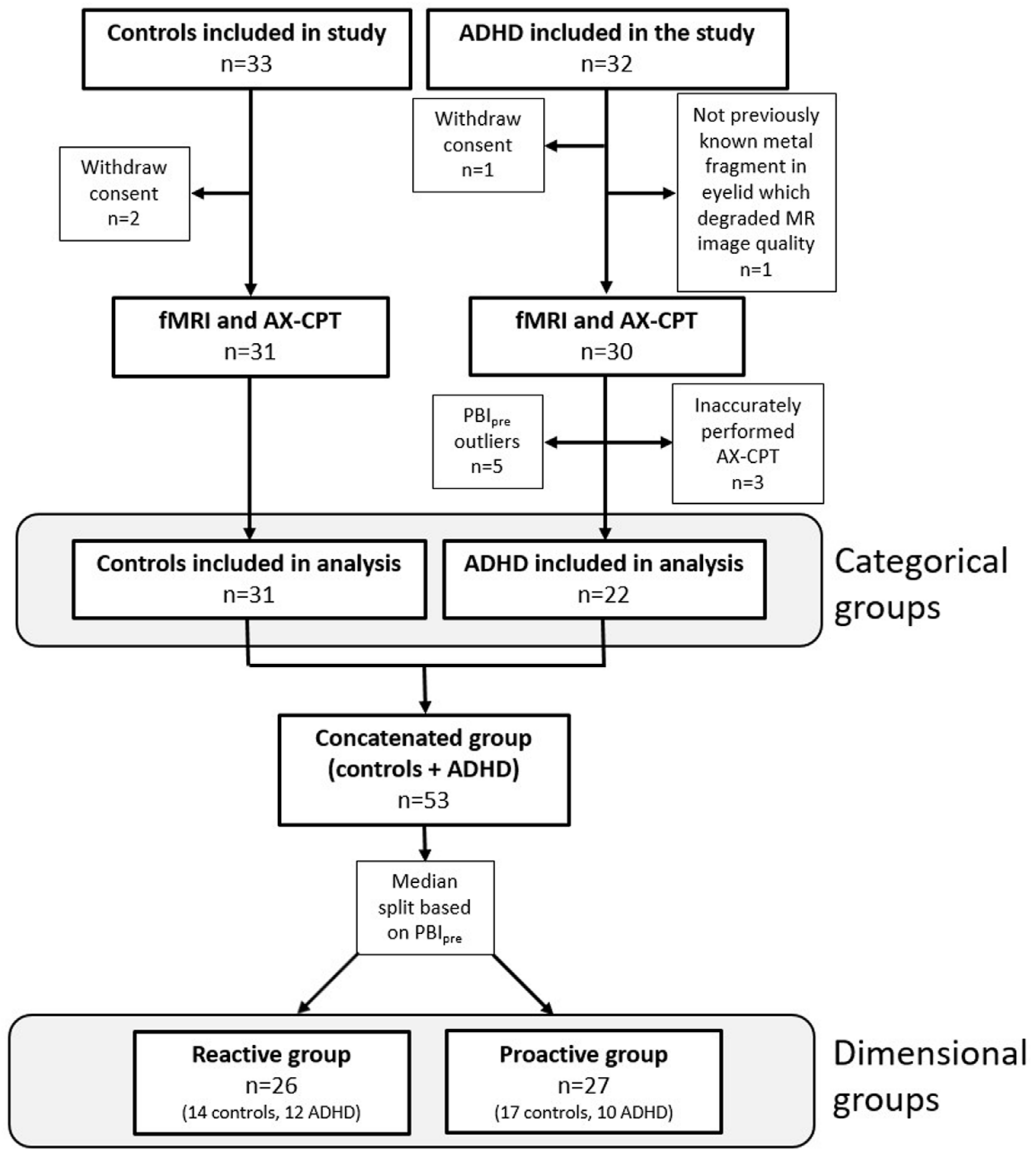
Flowchart showing inclusion and exclusion of participants in the study and the number of participants in the categorical and dimensional groups. Three participants from the ADHD group were excluded since PBI could not be calculated because they inaccurately performed the AX-CPT.

Of the 65 initial participants, 53 were included in the final analysis, which consisted of 31 healthy controls and 22 patients with ADHD (Figure 1). Five outliers were identified, and their experimental protocol carefully reviewed for any outstanding issue; in all cases circumstances could be identified that explained the extreme values and justified the exclusion of the participants. For the dimensional stratification, there were 26 individuals in the reactive group consisting of 14 healthy controls and 12 ADHD patients and 27 individuals in the proactive group consisting of 17 healthy controls and 10 ADHD patients. There was no significant difference in the number of healthy controls and ADHD patients clustering to the reactive and proactive groups (Pearson chi-square, χ(1) = 0.45, p = 0.50). Baseline and behavioral data for all participants are shown in Table 1.

**TABLE 1:** Baseline characteristics of study participants in the two stratification groups. Values are presented as mean ± 1 standard deviation. PBI is calculated using RT for AY and BX as PBI = (AY-BX)/(AX + BX).

		All (n = 53)	Controls (n = 31)	ADHD (n = 22)	Comparison (Controls vs. ADHD)	Reactive (n = 26)	Proactive (n = 27)	Comparison (Reactive vs. Proactive)
Age (years)		36 ± 10	37 ± 11	36 ± 9	p = 0.914	37 ± 11	36 ± 9	p = 0.689
Sex^a^ (Male/Female)		23/30	14/17	9/13	p = 0.86	15/11	8/19	p = 0.04
PBI_pre_ (a.u.)		0.15 ± 0.08	0.15 ± 0.07	0.15 ± 0.09	p = 0.957	0.09 ± 0.04	0.21 ± 0.05	p < 0.001 (due to median split)
PBI_post_ (a.u.)		0.17 ± 0.09	0.17 ± 0.09	0.17 ± 0.09	p = 0.971	0.14 ± .0.08	0.19 ± 0.09	p = 0.011
RT pre CS (msec)	AX	530 ± 139	520 ± 125	544 ± 158	p = 0.639	573 ± 155	488 ± 108	p = 0.013
	AY	631 ± 172	601 ± 134	673 ± 211	p = 0.170	665 ± 187	598 ± 153	p = 0.117
	BX	473 ± 165	457 ± 159	495 ± 174	p = 0.357	564 ± 180	385 ± 82	p < 0.001
	BY	462 ± 178	437 ± 143	499 ± 216	p = 0.209	519 ± 182	408 ± 158	p = 0.003
RT post CS (msec)	AX	505 ± 153	506 ± 147	503 ± 164	p = 0.986	534 ± 158	476 ± 145	p = 0.064
	AY	585 ± 169	588 ± 156	582 ± 190	p = 0.718	610 ± 173	561 ± 166	p = 0.24
	BX	433 ± 198	434 ± 188	431 ± 215	p = 0.871	473 ± 191	393 ± 199	p = 0.012
	BY	425 ± 190	422 ± 170	429 ± 220	p = 0.986	463 ± 177	388 ± 198	p = 0.013

CS, central stimulant medication;

a.u., arbitrary unit.

^a^ Sex assigned at birth.

### 3.2 Cognitive control paradigm (AX-CPT)–behavioral data

**FIGURE 2:**
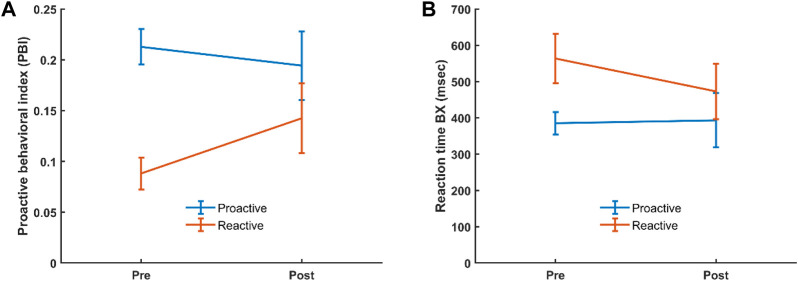
Interaction for PBI **(A)** and BX RT **(B)** in the reactive and proactive cognitive control mode groups, before and after administration of CS. Error bars show 95% confidence interval.

The AX-CPT used in the current study resulted in the expected behavioral features, and as previously reported in the literature AY trials showed the longest reaction time and highest error rate (Table 1; Supplementary Figure S1). For the behavioral data, we analyzed potential differences in reaction time (RT) and proactive behavioral index (PBI) in the categorical and dimensional groups before and after administration of CS by evaluating interaction effects. For the categorical stratification, we found no significant interaction regarding RT or PBI between healthy controls and ADHD patients before and after CS. For the dimensional stratification, however, a 2 × 2 repeated measure ANOVA showed a significant (F (1,51) = 8.777, *p* = 0.005) interaction for PBI where the reactive group significantly increased its mean PBI (*p* = 0.006) compared to the proactive group, which did not show any significant change in PBI, although there was a slight decrease in this (Figure 2A). Also, there was a significant pre-post CS interaction in RT for BX [F (1,51) = 4.516, *p* = 0.038], where the reactive (*p* = 0.004) but not proactive group significantly decreased its RT for the BX trial (Figure 2B).

### 3.3 Functional brain activation data

Similar to the behavioral data, we analyzed the fMRI data for interaction effects in order to assess potential brain activation differences between groups in the categorical and dimensional stratifications before and after administration of CS. The AY and BX trials were used as regressors, as they are the basis for calculating PBI and determining reactive and proactive cognitive control mode groups.

**FIGURE 3:**
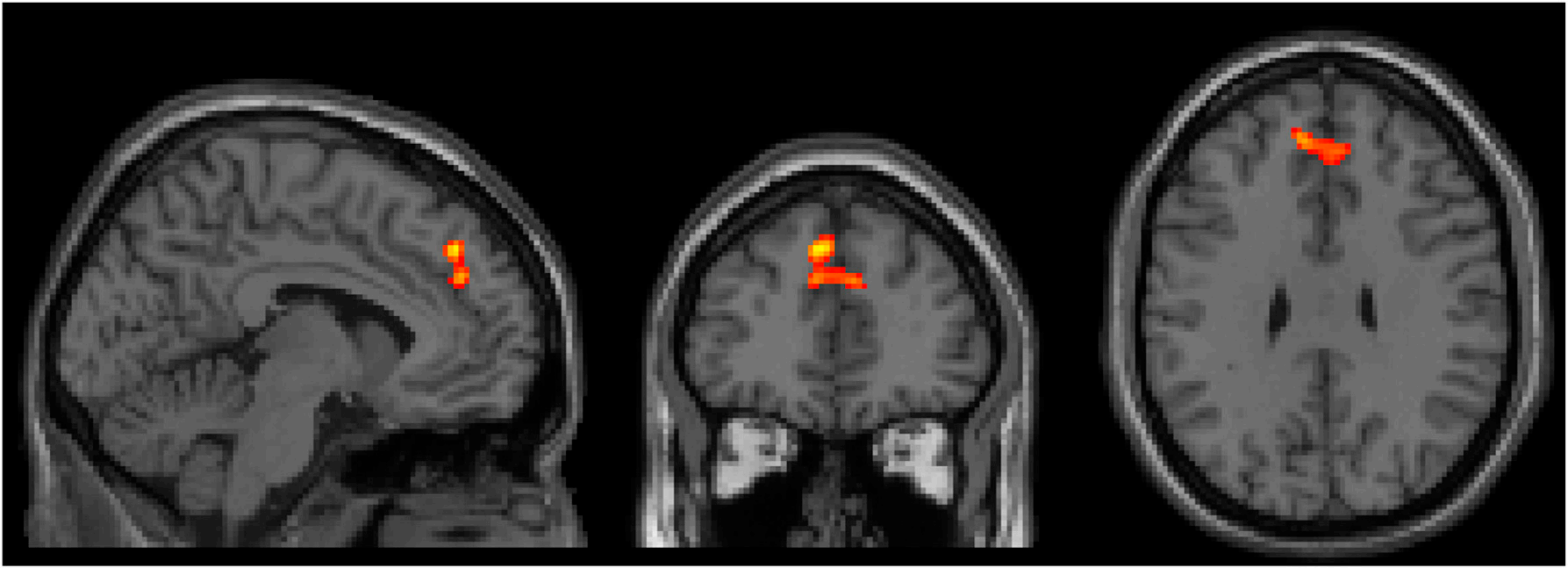
Three orthogonal slices (sagittal, coronal and axial) showing brain area where there was a significant increase of brain activity after CS for the reactive group compared to the proactive group. Images are shown using neurological orientation, i.e., subject left to the left in image.

There were no significant interaction effects between healthy controls and patients with ADHD before and after administration of CS in the categorical stratification. For the dimensional stratification, however, there was a significant interaction effect for BX. This interaction was noticed in a cluster located in the superior frontal and paracingulate gyri, showing that in these areas the reactive group increased its brain activation more after CS compared to the proactive group. The cluster consisted of 167 voxels (voxel size = 2 × 2 × 2 mm^3^) with a peak t-statistic coordinate at (−4, 42, 40 mm) in MNI-space (Figure 3).

**FIGURE 4:**
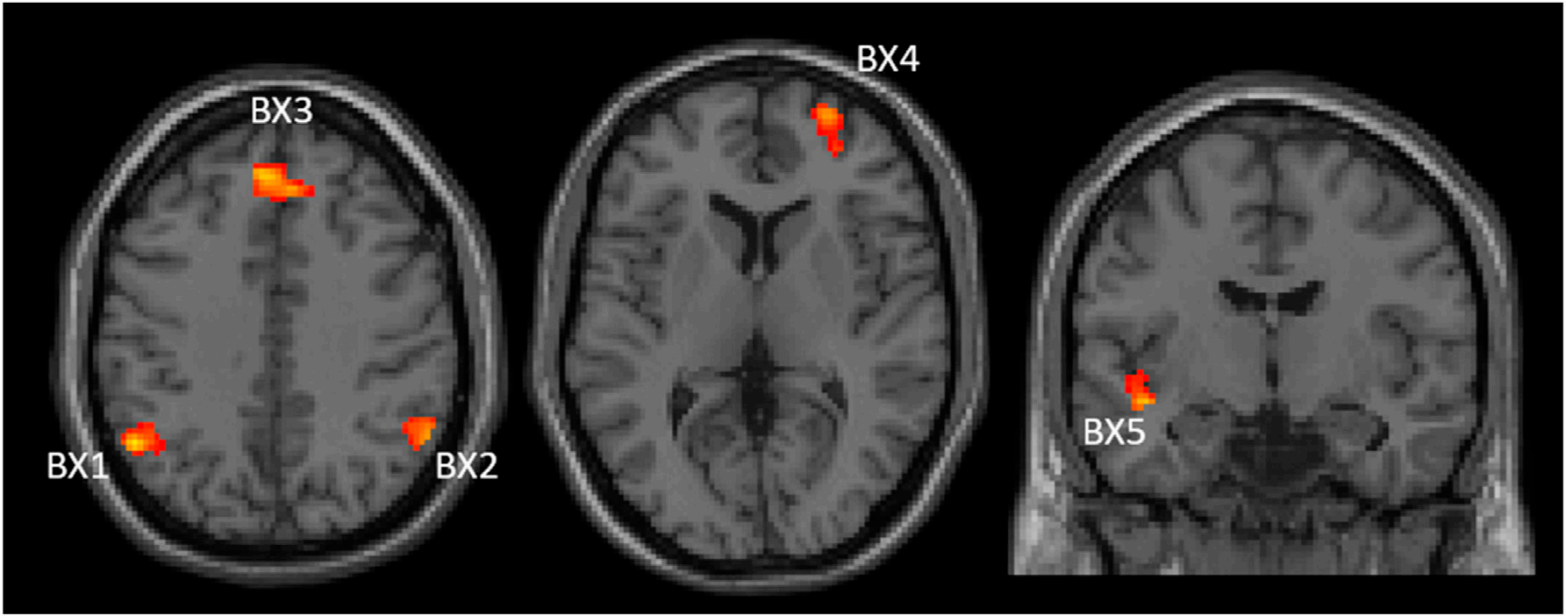
Two axial (left and middle) and one coronal (right) images showing significant clusters correlations between changes in brain activities (post-pre administration of CS) and PBI_pre_ during BX trials. The clusters are labelled (BX1-BX5) to be used for identification in text and Supplementary table S1 Images are shown using neurological orientation.

Correlation between baseline PBI (PBI_pre_) and changes in brain activation for the AY and BX trials after CS was also evaluated considering all participants as one group (n = 53). There were four significant clusters showing a negative correlation between PBI_pre_ and changes in brain activation after CS for the BX trial (BX1-BX4), and one cluster (BX5) showing a positive correlation (Figure 4). We saw no significant correlations between PBI_pre_ and changes in brain activation after CS for the AY trial. A summary of all displayed clusters shown in Figure 4 is provided in Supplementary Table S1 together with anatomical information about clusters.

**FIGURE 5:**
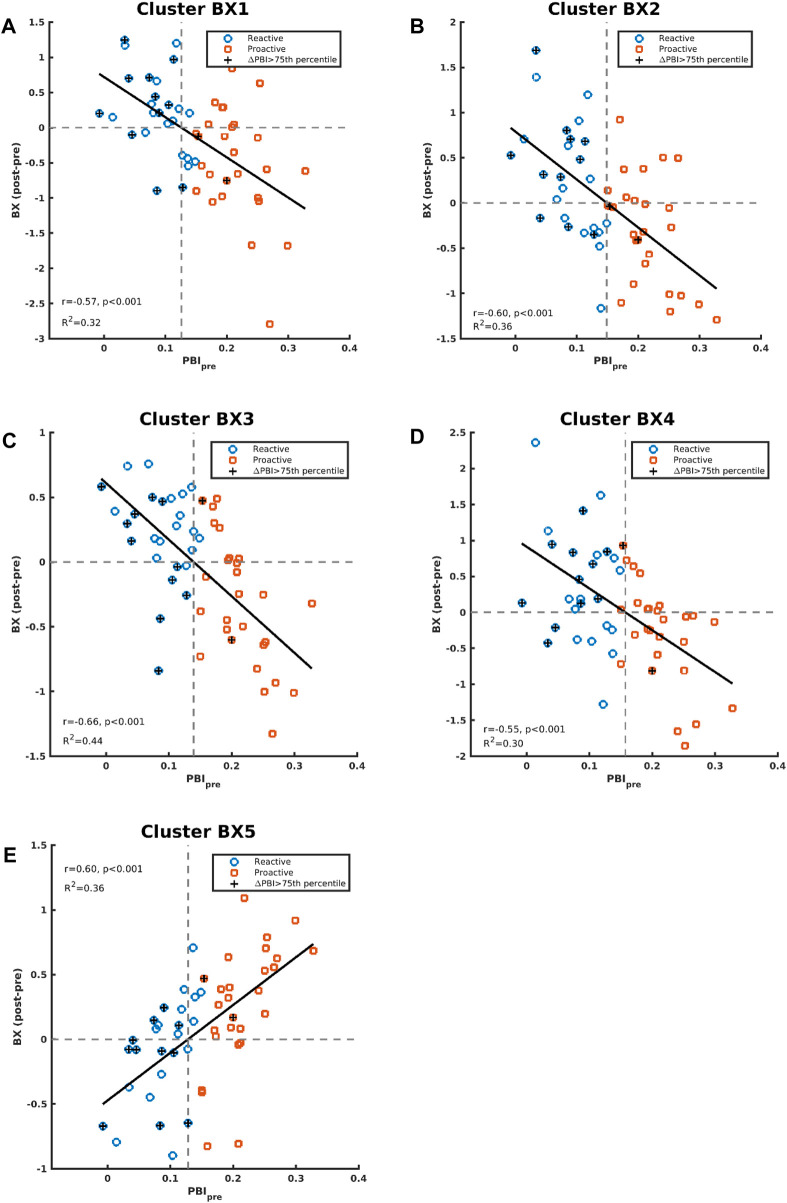
Correlations between contrast differences for BX (post-pre administration of CS) and PBI_pre_ are shown in panels **(A–E)** for cluster BX1-BX5, respectively. The individuals in the whole study who had an increase of PBI (PBI_post vs_ PBI_pre_) greater than the 75th percentile are indicated with “+”.

Figure 5 shows the correlations between PBI_pre_ and BX1-BX5 activation clusters. BX3 is virtually the same cluster obtained when comparing CS effects between the reactive and proactive groups in the dimensional stratification (Figure 3). The negative correlation indicates that reactive individuals increase brain activation in these areas after CS compared to proactive individuals, who show a decrease in brain activation after CS. For illustrative purposes, the individuals with largest increase in PBI after CS (defined as greater than the 75th percentile) are indicated in Figure 5. The majority of these individuals (11 out of 13) belong to the reactive group and most have an increase in brain activation after CS in clusters BX1-BX4 (BX1: 8/13, BX2: 8/13, BX3: 7/13, BX4: 10/13). In cluster BX5 the majority (8/13) decreased brain activity after CS. The regression lines in Figure 5 cross the *x*-axis at PBI_pre_ equal to 0.13/0.15/0.14/0.16/0.13 for clusters BX1-BX5, respectively.

## 4 Discussion

Psychiatric disorders are in almost all instances complex syndromes with as yet not fully established etiopathology (Insel, 2010). Diagnostic classification in clinical settings is guided by the presence and absence of characteristic phenomenological criteria that in themselves remain agnostic as to the neurobiological underpinning of the disorders (American Psychiatric Association, 2013). Lately, concern has been raised pertaining to a putative lack of reproducibility in the psychiatry/psychology research fields (Open Science Collaboration, 2015). Diagnostic heterogeneity has been postulated as one possible reason behind this (Borgogna et al., 2023). The utility of these classification systems for research purposes has thus been questioned, among other things, due to the substantial within group heterogeneity between group overlap the current categorical classification systems lead to (Widiger and Samuel, 2005; Wardenaar and de Jonge, 2013; Borgogna et al., 2023). The research domain criteria initiative (RDoC), on the other hand, although not a diagnostic tool *per se*, provides a neurobiologically informed dimensional framework for conceptualizing psychiatric disorders by utilizing neurobehavioral domains of function (Insel et al., 2010). Thus, dimensional stratification by reducing within group heterogeneity (Borgogna et al., 2023) may contribute to the standardization of a study population in a such a way that intervention, control and outcome (PICO) may become reproducible when similar PICOs are studied in different research facilities. The RDoC domains that are thought to be most relevant for ADHD are cognitive control and positive valence domains, pertaining to inattention and hyperactivity/impulsivity aspects of the disorder, respectively. Paradigms that evaluate these domains include the AX Continuous Performance Task (AX-CPT) for cognitive control domains such as goal selection, goal maintenance and updating (Lopez-Garcia et al., 2016), the Go NoGo task for response selection and response inhibition (Boucher et al., 2007), and various incentive delay tasks for positive valence domains such as reward anticipation (Knutson et al., 2000).

In this study, neural and behavioral effects of CS medication during a cognitive control task (AX-CPT) were investigated using two classification strategies to stratify study participants, (i) categorical stratification using DSM 5 diagnosis and (ii) dimensional stratification using RDoC cognitive control domain irrespective of diagnosis status. Median split of proactive behavioral index (PBI) calculated from AY and BX trials of the AX-CPT (Gonthier et al., 2016) was used to divided participants into reactive and proactive groups for the dimensional stratification without paying attention to diagnosis. Based on previous studies that found the effect of CS on cognitive control to vary with baseline cognitive capacity (Mattay et al., 2000; van der Schaaf et al., 2013; Rostami Kandroodi et al., 2021), we hypothesized that dimensional stratification may be superior to categorical stratification in revealing neural and behavioral effects of CS. Also, in the categorical stratification, we expected the effects of CS to be more prominent in ADHD patients compared to healthy controls, based on previous findings that showed a robust clinical effect of CS in ADHD patients (Faraone and Glatt, 2010; Cortese et al., 2018) and a less clear-cut cognitive enhancing effects in the non-clinical population (Repantis et al., 2010; Smith and Farah, 2011).

There was, of course, the possibility that the two stratification methods would result in largely similar individuals clustering in the same groups, in a way that would undermine our study design, i.e., that most ADHD patients would cluster into the reactive group and most healthy controls into the proactive group and that there would be no meaningful difference between the two stratification methods. Contrary to our initial apprehension, however, we found an almost 50/50 clustering of patients and healthy controls into the two dimensional groups, with the median split resulting in 14 healthy controls (45.2%) and 12 ADHD patients (54.5%) clustering in the reactive group and 17 healthy individuals (54.8%) and 10 ADHD patients (45.5%) clustering in the proactive group, with no significant difference in this between healthy controls and ADHD patients. The fact that cognitive control mode cuts across diagnosis indicates that ADHD patients may not markedly differ from healthy controls in this respect, unlike, for example, psychosis patients who have been shown to employ a markedly reactive cognitive control mode compared to healthy individuals (MacDonald, 2008).

Once it was ascertained that the stratification method gave variable clustering of individuals into the categorical and dimensional groups, we went ahead to systematically evaluate the effects of CS on the groups within the two stratification strategies. When participants were categorically assigned as healthy controls and ADHD patients, on the basis of DSM 5 diagnosis, we found no significant behavioral or brain activation effects of CS in these two groups. On the other hand, when study participants were stratified along dimensional factors into reactive and proactive cognitive control mode groups ignoring diagnosis status, CS selectively increased activation in paracingulate and superior frontal gyri in the reactive group compared to the proactive group and induced a shift towards proactive control mode in the reactive group without significantly affecting the proactive group. The shift towards proactive control mode in the reactive group was mediated by a decrease in reaction time for BX trials, without significant concomitant effects on AY trials. Besides these direct and selective effects of CS on brain activation and proactive behavioral index (PBI), we also found a significant correlation between baseline PBI and the neural effects of CS. In 4 out of the 5 active frontoparietal clusters for the BX contrast, lower baseline PBI was associated with greater post CS increase in brain activation. Clusters in frontal (frontal pole, bilateral paracingulate, right superior frontal gyrus) and parietal (bilateral angular and left supramarginal gyri) brain areas all showed increased brain activation after CS that was negatively correlated with baseline PBI scores. One left-lateralized temporo-insular cluster consisting of left insular cortex and left planum polare showed CS induced activation changes that were positively correlated to baseline PBI, i.e., the post-CS increase in these two areas was greater the higher baseline PBI score was.

Our results show that the neural and behavioral effects of CS were more clear-cut when participants were stratified along dimensional factors than diagnostic categories. In a recent study that included ADHD patients who responded and did not respond to CS treatment, we reported that saliency (“wanting”) and hedonic experience (“liking”), pertaining to the positive valence RDoC domain, could predict response to CS treatment and that that the scores for “wanting” were positively correlated to resting state connectivity increase in the ventral striatum (Rode et al., 2023). Although all included patients in the present study were clinical responders to CS treatment as evaluated by clinician- and patient-rated clinical global impression–improvement (CGI-I), our results from this study indicate that cognitive control mode might also be a potential predictor of CS response in clinical the setting. In a more recent paper Hung et al., 2024 reported that striatal structural connectivity and higher pre-treatment working memory scores were correlated with greater response to CS medication in patients with ADHD (Hung et al., 2024).

As mentioned above, the effect of CS on behavioral or neural outcomes varies with baseline cognitive capacity (Mattay et al., 2000; van der Schaaf et al., 2013; Rostami Kandroodi et al., 2021), baseline DA and NA levels (Cools and D'Esposito, 2011) and rate of behavioral, physical or electrical stimulation (Sanger and Blackman, 1976). Because the proactive group had substantially lower mean RT scores for, among other things, BX and BY trials, if the proactive group were to be allowed to respond at a higher rate, this could potentially affect the results due to the rate dependency of CS effect (Sanger and Blackman, 1976). However, in the present paper, the rate of stimulus (interstimulus interval) across all subjects and conditions was constant. Thus, even though a participant had a shorter RT, this was not allowed to alter the rate of response. Furthermore, due to the low mean RT scores in the proactive group, their response to CS as far as RT is concerned could be restricted due to ceiling effects. However, when we looked at baseline RT scores across all participants, we found that both proactive and reactive individuals had baseline RT scores that ranged from low to high and did not cluster around any particular value in a way that could have imposed significant ceiling effects.

A plausible clinical implication based on the results in the current study might be that ADHD patients employing reactive cognitive control mode, as well as healthy controls who might use CS off-label, would respond better to CS than those employing a more proactive control mode. This assumes that a transition from reactive to proactive control mode is equated to being a responder of CS. Further studies are needed to elucidate this and should include groups of both CS responding and CS non-responding ADHD patients.

The lack of effect of CS in unstratified ADHD patients and unstratified healthy controls, we suspect, might be due to substantial within group heterogeneity and the known inverted U-form dose-effect curve of the signal substances dopamine and noradrenaline whose peri-synaptic levels are enhanced by CS medication. Similar lack of effect was previously reported in unstratified study participants (Cools et al., 2008), and the CS effects could be revealed when participants were stratified along baseline cognitive capacity (Cools et al., 2008) or baseline dopamine synthesis capacity (Westbrook et al., 2020).

Our implementation of the AX-CPT paradigm in this study corresponds to a low load task (Mäki-Marttunen, Hagen, and Espeseth, 2019), since it only consisted of two cue letters (“A” and “B”) and two probes (“X” and “Y”) and that the intra-trial interval was short. The behavioral and neural effects of the task can be expected to be larger when using a more challenging version with more cues and probes and longer intra-trial interval. However, we found such versions too difficult for some of the ADHD patients that could have led to an even greater loss of included ADHD participants, which occurred even when using this easier version (see Figure 1).

### 4.1 Limitations

In this study we included only ADHD patients that have clinically been judged to be responders to CS treatment. It is theoretically possible that the results may have been different had we also included ADHD patients who were CS non-responders. Another intriguing question is why ADHD patients who have clinically responded to CS medication become “non-responders” when tested with a cognitive control paradigm, if they happen to employ proactive cognitive control mode. One possible explanation for this is suggested by the recent finding of (Bowman et al., 2023), who in healthy subjects found that CS increased motivation/effort but reduced quality of effort, which suggests that CS responding ADHD patients might improve on certain but not all aspects of their impairment.

## 5 Conclusion

We can draw several conclusions from the current study, (i) cognitive control mode cuts across diagnostic categories and there is equal likelihood for ADHD patients to employ reactive and proactive cognitive control mode as healthy controls, (ii) dimensional stratification under our experimental condition seems to be superior to categorical stratification in revealing neural and behavioral effects of CS, and (iii) baseline cognitive control mode might potentially be a predictor of CS treatment effect in the clinical setting.

## Data Availability

The raw data supporting the conclusions of this article will be made available by the authors, without undue reservation.
