# Supplementary material for: Categorical and dimensional aspects of stimulant medication effects in adult patients with ADHD and healthy controls
Source: Front Pharmacol. 2024 Jul 10;15:1412178. doi: 10.3389/fphar.2024.1412178 (PMC11266130; doi:10.3389/fphar.2024.1412178)
Supplement: Supplementary file 1 [file DataSheet1.docx]

# **Supplement to** Categorical and dimensional aspects of stimulant medication effects in adult patients with ADHD and healthy controls

## Preprocessing of fMRI images and quality control

Acquired AX-CPT fMRI images were preprocessed using CONN (release 20.b) and included the following steps:

- realignment and unwarping of fMRI images using SPM12 *realign & unwarp* (Andersson et al. 2001)
- slice-timing correction (STC) using SPM12 (Henson et al. 1999). Time stamping for the pulse sequence was obtained from scanner.
- segmentation and normalization (structurals target resolution = 1mm and functionals target resolution = 2mm) using SPM12 procedures (Ashburner and Friston 1997)
- outlier detection (framewise displacement above 0.9mm or global BOLD signal changes above 5 s.d. are flagged as potential outliers)
- smoothing using SPM12 (kernel FWHM = 6 mm)

Standard settings in CONN were applied except smoothing where a kernel FWHM of 6 mm was used instead of 8 mm. An overview (montage) of both structural and functional images after preprocessing was inspected in order to ensure correct normalization to MNI-space. The number of detected outliers were counted for each participant in pre and post, respectively. The highest number of outlier volumes in a single AXCPT-acquisition was 10%, which was considered as acceptable.

After preprocessing the denoising pipeline in CONN was applied which was based on an anatomical component-based noise correction procedure (aCompCor) (Behzadi et al. 2007) and included:

- noise components from white matter and cerebrospinal areas
- estimated subject-motion parameters (from realignment procedure)
- scrubbing (from outlier detection procedure)
- a band-pass filter [0.008 – Inf Hz]

Preprocessed and denoised image volumes (named ‘dswau… .nii’ in CONN) were used in the first level analysis.

A thorough description of preprocessing and denoising using CONN can be found in (Morfini, Whitfield-Gabrieli, and Nieto-Castanon 2023) and at https://web.conn-toolbox.org/home.

## Behavioral performance during AX-CPT

Response times and error rates for all trials pre and post intervention are shown in Fig. S1. The reaction time and error rate is highest for AY trials which is in agreement with previous studies (Lopez-Garcia et al. 2016; Gonthier et al. 2016). Our implementation of AX-CPT results in low error rates and can be considered as a low load version of AX-CPT (Mäki-Marttunen, Hagen, and Espeseth 2019).


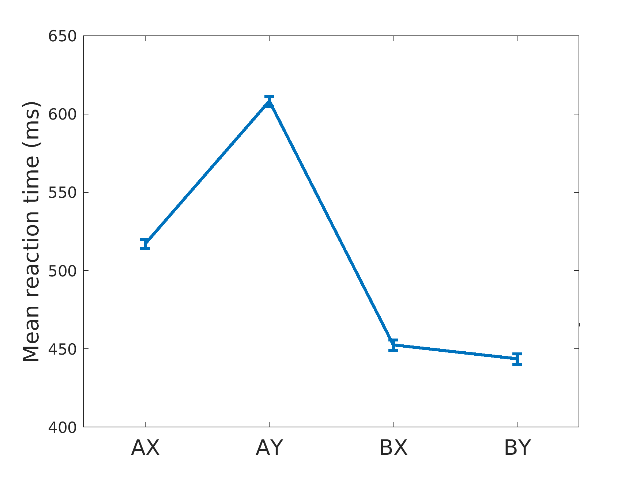

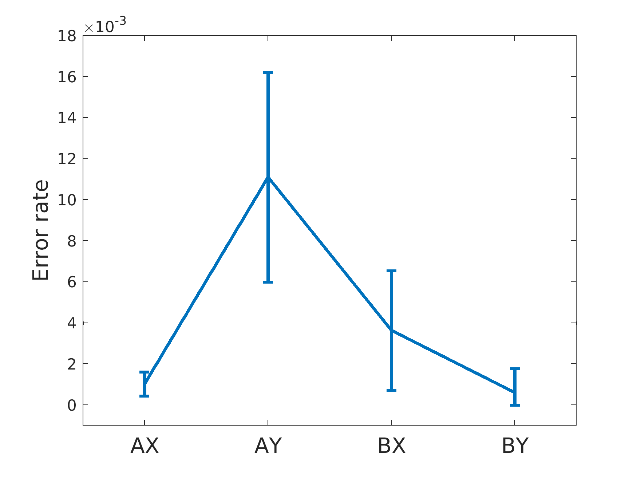


1. (b)

**Figure S1.** (a) Reaction times and (b) error rates for the four trials (AX, AY, BX, BY) using merged pre and post data. Error bars show 95% confidence intervals.

The number of AY and BX responses for all participants is shown in Figure 2. The number of AY and BX trials was always 16. The number of omissions is thus represented by the difference between the number of trials and the number of responses. The five outliers are indicated with black circles around the individual’s AY-trial

(a)
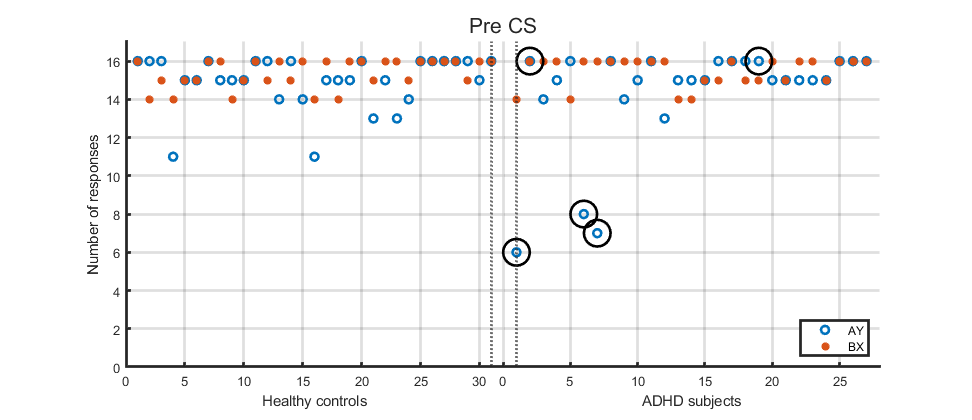


(b)
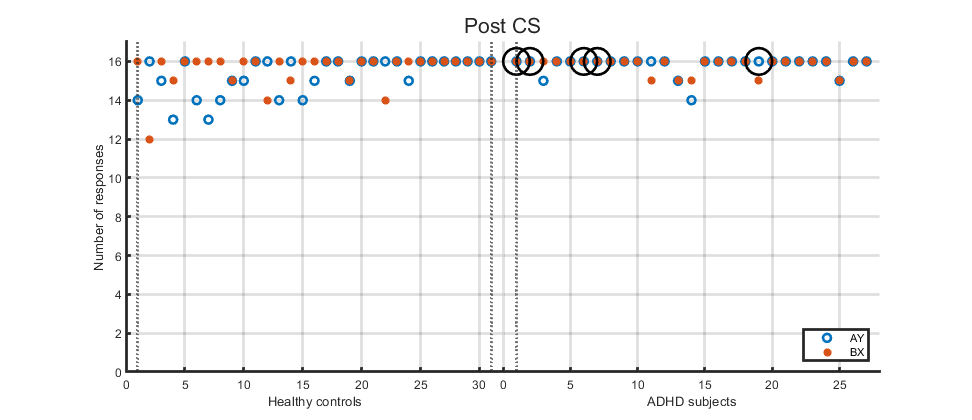


**Figure S2.** The number of responses for the AY and BX trials for all healthy controls (n=31) and ADHD patients (n=27) performing AX-CPT (a) pre and (b) post central stimulant (CS) administration. The five outliers are indicated with black circles around the individual’s AY trials.

## Anatomical information for clusters BX1-BX5

Anatomical information about the five clusters in Figure 4 (BX1-BX5) main text is provided in Table S1.

| **Cluster** | **Anatomical location (cluster)** | **Hemisphere** | **Size (coordinate of peak t-statistic)** |
| --- | --- | --- | --- |
| BX1 | Angular gyrus  Lateral occipital cortex Supermarginal gyrus, posterior division | left | 177 (-50, -56, 42) |
| BX | Angular gyrus | right | 243 (56, -52, 36) |
| BX3 | Superior frontal gyrus  Paracingulate gyrus | left/right | 281 (-2, 40, 42) |
| BX4 | Frontal pole | right/left | 96 (28, 60, 6) |
| BX5 | Planum polare  Insular cortex | right | 97 (-42, -6, -12) |

**References**

Andersson, J. L., C. Hutton, J. Ashburner, R. Turner, and K. Friston. 2001. 'Modeling geometric deformations in EPI time series', *Neuroimage*, 13: 903-19.

Ashburner, J., and K. Friston. 1997. 'Multimodal image coregistration and partitioning--a unified framework', *Neuroimage*, 6: 209-17.

Behzadi, Y., K. Restom, J. Liau, and T. T. Liu. 2007. 'A component based noise correction method (CompCor) for BOLD and perfusion based fMRI', *Neuroimage*, 37: 90-101.

Gonthier, C., B. N. Macnamara, M. Chow, A. R. Conway, and T. S. Braver. 2016. 'Inducing Proactive Control Shifts in the AX-CPT', *Front Psychol*, 7: 1822.

Henson, Richard , C Buechel, O Josephs, and Karl Friston. 1999. 'The slice-timing problem in event-related fMRI', *NeuroImage 9(6)*: 125.

Lopez-Garcia, P., T. A. Lesh, T. Salo, D. M. Barch, A. W. MacDonald, 3rd, J. M. Gold, J. D. Ragland, M. Strauss, S. M. Silverstein, and C. S. Carter. 2016. 'The neural circuitry supporting goal maintenance during cognitive control: a comparison of expectancy AX-CPT and dot probe expectancy paradigms', *Cogn Affect Behav Neurosci*, 16: 164-75.

Morfini, F., S. Whitfield-Gabrieli, and A. Nieto-Castanon. 2023. 'Functional connectivity MRI quality control procedures in CONN', *Frontiers in Neuroscience*, 17: 1092125.

Mäki-Marttunen, V., T. Hagen, and T. Espeseth. 2019. 'Task context load induces reactive cognitive control: An fMRI study on cortical and brain stem activity', *Cogn Affect Behav Neurosci*, 19: 945-65.
